# Second surgery for progressive glioblastoma: a multi‐centre questionnaire and cohort‐based review of clinical decision‐making and patient outcomes in current practice

**DOI:** 10.1007/s11060-021-03748-0

**Published:** 2021-03-31

**Authors:** P. M. Brennan, R. Borchert, C. Coulter, G. R. Critchley, B. Hall, D. Holliman, I. Phang, S. J. Jefferies, S. Keni, L. Lee, I. Liaquat, H. J. Marcus, S. Thomson, L. Thorne, M. Vintu, A. N. Wiggins, M. D. Jenkinson, S. Erridge

**Affiliations:** 1 Translational Neurosurgery, Centre for Clinical Brain Sciences, University of Edinburgh, Chancellor’s Building, Edinburgh BioQuarter, 49 Little France Crescent, Edinburgh, EH16 4SB UK; 2 Addenbrookes University Hospital, Cambridge, UK; 3 Royal Victoria Hospital, Newcastle, UK; 4 Brighton and Sussex University Hospitals NHS Trust, Brighton, UK; 5 Institute of Translational Medicine, University of Liverpool, Liverpool, UK; 6 Department of Neurosurgery, The Walton Centre NHS Foundation Trust, Liverpool, UK; 7 Lancashire teaching Hospitals, Preston, UK; 8 University of Edinburgh medical School, Edinburgh, UK; 9 Department of Clinical Neuroscience, NHS Lothian, Edinburgh, UK; 10 National Hospital for Neurology and Neurosurgery, Queen Square, London, UK; 11 Leeds Teaching Hospitals, Leeds, UK; 12 University College London Hospitals, London, UK

**Keywords:** Glioblastoma, Progression, Recurrence, Surgery

## Abstract

**Purpose:**

Glioblastoma prognosis is poor. Treatment options are limited at progression. Surgery may benefit, but no quality guidelines exist to inform patient selection. We sought to describe variations in surgical management at progression, highlight where further evidence is needed, and build towards a consensus strategy.

**Methods:**

Current practice in selection of patients with progressive GBM for second surgery was surveyed online amongst specialists in the UK and Europe. We complemented this with an assessment of practice in a retrospective cohort study from six United Kingdom neurosurgical units. We used descriptive statistics to analyse the data.

**Results:**

234 questionnaire responses were received. Maintaining or improving patient quality of life was key to decision making, with variation as to whether patient age, performance status or intended extent of resection was relevant. *MGMT* methylation status was not important. Half considered no minimum time after first surgery. 288 patients were reported in the cohort analysis. Median time to second surgery from first surgery 390 days. Median overall survival 815 days, with no association between time to second surgery and time to death (p = 0.874).

**Conclusions:**

This is the most wide-ranging examination of contemporaneous practice in management of GBM progression. Without evidence-based guidelines, the variation is unsurprising. We propose consensus guidelines for consideration, to reduce heterogeneity in decision making, support data collection and analysis of factors influencing outcomes, and to inform clinical trials to establish whether second surgery improves patient outcomes, or simply selects to patients already performing well.

**Supplementary Information:**

The online version contains supplementary material available at 10.1007/s11060-021-03748-0.

## Introduction

Glioblastoma (GBM), the most common primary intracranial malignancy, represents 30% of all central nervous system tumours and has an incidence of 3.22 per 100,000 individuals [1]. Standard care for newly diagnosed GBM is maximal safe resection, radiotherapy and concurrent and adjuvant temozolomide, and has changed little in 15 years [2]. Despite treatment, prognosis is poor, with a median overall survival of 15 months. Tumour progression invariably occurs and may be referred to as recurrence [3]. Tumour progression nearly always develops within the margin of the original tumour [4]. At the time of tumour progression, clinicians may opt for best supportive care, further surgical excision, irradiation, chemotherapy or a combination of the above [5]. Survival following non-surgical interventions varies widely [6]. Given the paucity of second line chemotherapy for the management of tumour progression, and the symptomatic consequences of tumour progression, there is an urgent need to determine the role of surgery [7].

Second surgical intervention was first proposed in 1968. It has been suggested that 25% of patients with progressive tumour may be eligible for consideration of second surgery [5]. However, the evidence base for this is poor. Studies that evaluated impact of second surgery have almost exclusively been based on single center retrospective cohorts, with no comparison to patients who do not get second surgery. Some of these studies reported greater overall survival and progression-free survival in select patients following second surgery [8–10]. A meta-analysis and systematic review suggested second surgery in select patients conferred a survival advantage [11]. However, a subsequent meta-analysis recognized that the effect of the timing of second surgery on survival had been ignored [12]. They demonstrated that there was no survival benefit following reoperation once time to second surgery was controlled for.

There is a lack of evidence-based guidelines to support identification of which patients might benefit from second surgery. The 2018 National Institute of Clinical Excellence (NICE) guidelines (NG-99) [13] for brain tumours offer advice on management options (surgical and chemotherapy) to consider in the treatment of disease progression, but no specific criteria by which to select patients to the various options. European Association of Neuro-oncology (EANO) guidelines simply suggest the option of second surgery should be explored, but without guidelines to support patient and clinician decision making [14]. Improved guidance for treatment selection would help patients and clinicians, reducing diversity in decision making, and would form a benchmark for the development of clinical trials and an improved evidence base.

The aim of this study was to determine areas of consensus and variation in practice, highlight where further evidence base needs to be developed, and build towards a consensus management of GBM progression. This will serve as a benchmark for new guidelines and intervention studies. To develop a thorough understanding of current practice in selection of patients with progressive GBM for surgery, we surveyed surgeons in the UK and Europe. To further define the role of surgery, and to assess the extent to which actual practice mirrored that reported in the survey, we analysed outcomes following surgical management of tumour progression in six United Kingdom neurosurgical units.

## Methods and materials

### Questionnaire of practice

A survey was designed using google docs (docs.google.com) to elicit details of current management by the neuro-oncology MDT (tumour board) of patients with GBM at the time of first treatment failure (Appendix 1).The survey was approved by the Society of British neurosurgeons (SBNS), the UK neuro-oncology community, and the European Association of Neurosurgeons (EANS). The survey was distributed by these organisations via email to their individual members, with online data collection. Respondents were asked to report the consensus management strategy for their local hospital MDT with regards to diagnosis of progression, decision making about proceeding to second surgery, and to ascertain what post-operative treatment was used. Data was analysed using descriptive statistics including measures of frequency such as count and percentage. Regional differences were outlined.

### Retrospective surgical cohort analysis

#### Study design

This was a multi-centre retrospective study in a cohort of patients with GBM progression who underwent second surgery between January 2011 and January 2017, at 6 neurosurgical units in the United Kingdom: Brighton, Edinburgh, Leeds, Newcastle, London, and Liverpool. Outcomes were assessed until October 31st, 2018.Local approval was obtained from each institution for anonymised data to be collected.

#### Participants & treatment characteristics

Patients eligible for this study: (i) had a histological diagnosis of GBM as determined by a consultant neuropathologist based on prevailing WHO classification (ii) received the Stupp protocol for treatment of primary disease (surgical resection, concurrent and adjuvant temozolomide and 60 Gy of fractionated radiotherapy iii) underwent at least one further instance of surgery for disease progression. Patients were identified from databases of surgical neuro-oncology patients maintained in each centre. Details including age at diagnosis and tumour location were recorded. Whether or not patients were offered adjuvant chemotherapy was also noted.

#### Outcomes

Overall survival was calculated from the date of histological diagnosis (i.e., date of first surgery) to date of death or last follow-up. Date of progression was defined from the date of second surgery.

Recorded adverse event data following all operations was collected and classified into (i) ‘neurological/pertaining to surgery’ (ii) non-neurological/systemic (iii) infection. Patients in whom a pre-operative neurological deficit existed and did not change following surgery were not classed as having had a neurological complication.

#### Statistical methods

SPSS v.24 was used to perform statistical analyses. Bivariate correlations were analysed using Pearson correlation. Threshold for significance was set as p-value ≤ 0.05.

## Results

### Questionnaire of practice

**Fig. 1:**
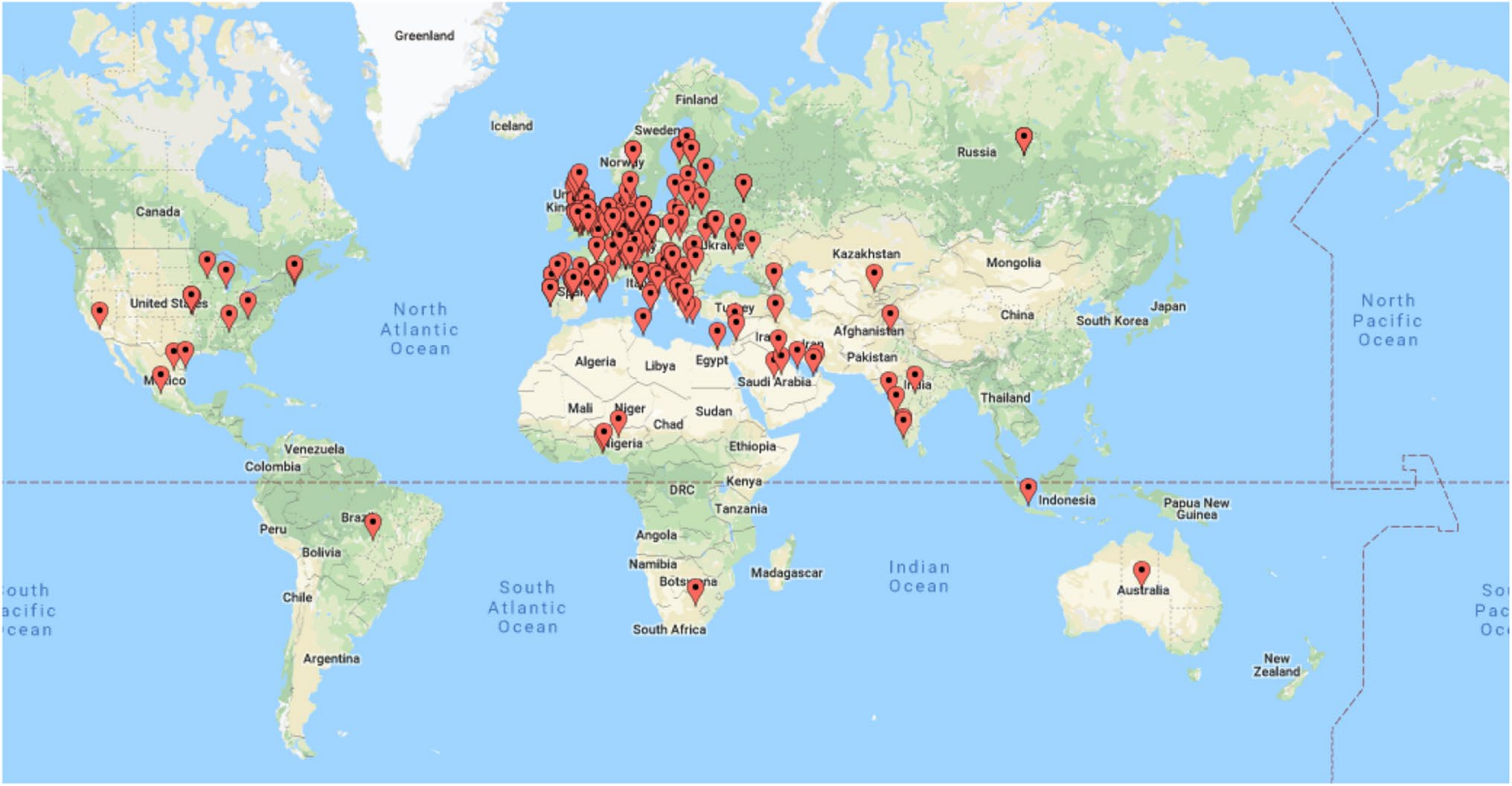
International distribution of respondents to online questionnaire

234 responses were received between January and March 2018. There were 45 responses from the UK, 124 from Europe and 65 from the rest of the world. The international distribution of responders is shown in Fig. 1.

139 (59.4%) responses were from neuro-oncology subspecialty neurosurgeons, 82 (35.0%) from neurosurgeons without a declared subspecialty interest in neuro-oncology, 12 (5.1%) responses were from clinical oncologists and 1 response (0.4%) was from a neurologist. We combined the analysis of surgeons and other clinicians in the neuro-oncology team to capture the spectrum of practice. The speciality of the clinician in charge of a patient at time of identification of progression could be a surgeon or oncologist.

We asked each respondent to record the number of craniotomies for GBM performed annually in each hospital, and the number of second surgeries for tumour progression. Broadly the number of second surgeries increased in proportion to the number of primary surgeries (Table 1), and the trends were similar between UK, Europe, and the rest of the world. (Table 2).

**Table 1:** Summary of the number of primary surgeries and second surgeries for glioblastoma according to questionnaire respondents

Number of GBM surgeries per year	Number of units*	Number of surgeries for GBM progression per year (n)		
		< 5	5–10	> 10
> 100	48	0	7	41
51–100	84	7	26	51
21–50	70	27	27	16
1–20	26	21	3	2

*Data point not answered by 6 responders

**Table 2:** Number of institutions by volume of primary and second surgeries stratified for UK, Europe and out-with Europe

Number of GBM surgeries per year	UK: number of institutions by primary and re-do surgeries per year (% of category)			Europe: number of institutions by primary and re-do surgeries per year (% of category)			Out-with Europe: number of institutions by primary and re-do surgeries per year (% of category)		
	< 5	5–10	> 10	< 5	5–10	> 10	< 5	5–10	> 10
> 100	0 (0)	4 (20)	16 (80)	0 (0)	3 (14)	18 (86)	0 (0)	0 (0)	7 (100)
51–100	1 (8)	3 (23)	9 (69)	5 (9)	16 (29)	34 (62)	1 (6)	7 (44)	8 (50)
21–50	3 (50)	2 (33)	1 (17)	17 (45)	15 (39)	6 (16)	7 (27)	10 (38)	9 (35)
1–20	1 (100)	0 (0)	0 (0)	8 (80)	2 (20)	0 (0)	12 (80)	1 (7)	2 (13)

#### Radiological surveillance, diagnostic criteria, and multidisciplinary review

Following completion of Stupp protocol treatment, 81.2% of responders reported performing radiological surveillance every 3-4 months in the first year. This decreased to 54.4% in the second year, with 37.6% instead opting for an imaging interval of 6 months. MRI was the most common modality used to diagnose progression and 94.4% respondents rely on identification of contrast enhancing disease, 44.0% on MR spectroscopy and 44.4% on MR perfusion. PET was also used for diagnosis by 26.1% of responders.

Interpretation of the significance of imaging changes often benefit from consideration of other patient factors and discussion within the multidisciplinary team. 68.4% of respondents presented every case of disease progression for MDT review (55.5% of respondents in the UK, 79.0% in Europe), 12.0% presented only those with progression local to the site of first surgery, and 7.3% only patients who had survived more than 9 months.

#### Patient and disease characteristics for consideration of surgery for progressive disease

##### Quality of life

More than 90% of respondents noted that maintaining and/or improving patient quality of life was a key determinant of the decision to proceed to surgery.

##### Age

44.9% of respondents did not consider that age influenced decision making, whilst 27.8% of respondents thought age over 80 years made the patient unsuitable for second surgery, and 24.4% if the patient’s age was over 70 years.

##### Performance status

When asked if there was a minimum KPS at the time of diagnosis of tumour progression for the patient to be considered for second surgery, 36.8% reported a minimum of 70, 23.5% a minimum of 80, 10.7% a minimum of 90 and only 14.5% reporting that performance status did not influence their decision making.

##### *MGMT* methylation status

A quarter (25.2%) of respondents reported that their units did not routinely test MGMT tumour status from the first surgery. Of the remaining respondents, 72.6% did not consider that *MGMT* methylations status should influence decision making.

##### Anticipated resection

Over half (56.8%) of respondents would only consider surgery for tumour progression if a greater than 90% extent of resection (EoR) of the enhancing component was anticipated. Only 17.1% responded that the anticipated EoR did not influence their decision making.

##### Timing after first operation

If tumour progression occurred whilst a patient was still being treated following the primary surgery, 45.7% of respondents thought this precluded further surgery. The time elapsed after first surgery to progression was thought to be important in decision making (62.8%), rather than the time after completion of chemotherapy. However, 45.3% did not consider there to be a minimum duration after the first operation before consideration of second surgery for disease progression. There was no consensus amongst the remainder of respondents. Once the decision for surgery for progressive disease has been made, 78.6% of respondents thought surgery should then be prioritised on the next available list.

#### Second line chemotherapy and re-irradiation

Following second surgery, 48.7% of respondents reported the multidisciplinary team would consider treatment with PCV, 41.9% with repeat temozolomide challenge if tumour methylated and 19.7% if unmethylated. 25.6% would consider single agent lomustine. Other therapies under consideration were bevacizumab (5.5%) or irinotecan (1.3%). 49.1% of respondents would consider re-irradiation, 20.9% would not, with the remainder being open to consider it in selected cases, especially in patient with longer survival from first surgery. 40.2% of respondents would consider second surgery even if there were no further oncological treatment available. For these questions, respondents could complete more than one option.

### Retrospective surgical cohort analysis

#### Patient demographics and tumour information

Across the 6 neurosurgical units, 288 patients were identified as having undergone second surgery for tumour progression during the study period. 24 patients (8.3%) had 3 operations. 67 (23.2%) of patients were alive at the time of study.

The median overall survival was 815 days (27.2 months) (IQR 547–1296 days). The median age was 51 years (IQR 44–60 years). The anatomical distribution of primary tumours was predominantly supratentorial, with 248 occupying a single lobe (101 temporal, 85 frontal, 51 parietal, 11 occipital, 1 thalamic, 1 tentorial, 1 intraventricular) and 34 spread across 2 lobes. Multifocal tumours at diagnosis were identified in 3 patients. Tumour laterality was relatively even, with 165 right sided tumours and 122 left sided. Only 1 patient in this cohort was documented as having bilateral disease.

#### Treatment characteristics for tumour progression

Median time to second surgery from first surgery was 390 days (13 months) (IQR 241–686 days). The median time to death following second surgery was 316 days (10.5 months) (IQR 174–595 days). There was no association between time to second surgery and time to death following second surgery (Pearson correlation 0.009; p = 0.874). However, greater time to reoperation was positively correlated with greater overall survival (Pearson correlation = 0.655; p < 0.001). 192 patients (81.7%) were offered further chemotherapy and 43 (18.3%) were not; 53 patients had no data as to whether they were offered further chemotherapy.

#### Complications following surgery

For patients with data on adverse events following second surgery, 54 (23.0%) experienced an adverse event; 35 (14.9%) neurological and 7 (3%) non-neurological excluding infection. This compared to 42 patients (17.4%) having an adverse event recorded at first surgery; 11.6% had neurological sequalae, 10 (4.1%) had non-neurological events excluding infection. Cranial infection rate for all patients at 1st, 2nd and 3rd surgery was 2.1%, 5.1 and 12.5% respectively.

## Discussion

Our questionnaire aimed to scope out a breadth of opinions and to identify what, if any, consensus exists. Such questionnaires provide only a snapshot of practice and there will be variations within countries, hospitals and even in the practice of individual clinicians that are not adequately delineated with this strategy. However, our analysis has captured the variation that exists in the clinic about both patient selection and choice of surgical management, for people with progressive GBM. In the absence of existing robust evidence-based guidelines, this is not surprising. A 2016 study of treatment algorithms for management of GBM progression in Switzerland identified considerable variability in the factors used for treatment decision making. ‘Fitness’, ‘resectability’ and time from first surgery were used as criteria for determining re-treatment, including surgery.

The missing data in our cohort analysis reflects the relative low rate of surgery for tumour progression and the long period over which it was necessary to analyse data. It is possible that the centres we surveyed may not be representative of UK neurosurgery, but our findings are comparable to other published data. In our analysis, like most published data, the outcomes for patients who do not undergo second surgery, matched for clinical features, was not available. We know which patients selected for surgery perform best, but not whether a second operation provides an independent survival benefit.

As expected, a post-contrast MRI is the mainstay investigation leading to determination of disease progression. The UK NICE NG-99 [13] guidelines recommend imaging every 3–6 months for the first 2 years after treatment, annually to year 4 and every 1-2 years thereafter. The questionnaire responses were consistent with this. The NG-99 guidelines, which were published after the questionnaire was undertaken, are based on expert consensus rather than an evidence base. In both the NG-99 guidelines and the questionnaire responses, frequency of imaging reduces as survival increases.

Advanced MRI techniques may increase diagnostic accuracy of disease progression [15], as compared to pseudo progression, but are difficult to interpret and the NG-99 guideline committee recommended them only where extra information was likely to substantially alter treatment plans. Fewer than 50% of questionnaire respondents reported using MR spectroscopy or perfusion, and we don’t know whether that was for specific diagnostic difficulty, or as a routine methodology.

With uncertainty about diagnostic criteria for determining disease progression, the collective experience and opinions of the MDT are invaluable. NG-99 guidelines recommend that ‘if people having active monitoring show radiological or clinical disease progression, discuss this at a multidisciplinary team meeting.’ In fact, only 68% of all patients with GBM progression are currently being discussed. This was lower in the UK (56%) compared to Europe (79%). The questionnaire responses indicated that in some cases MDT referral was only made if survival since diagnosis was more than 9 months or where disease progression was localised, neither of which variables has been well evidenced to be predictive of superior prognosis in response to further therapy.

In our retrospective cohort, overall median survival in the cohort study was 815 days, but without a comparator of non-operative patients, the true merits of surgery cannot be ascertained. The median interval between first and second surgery was 13 months, suggesting a preference to select better performing patients. Significantly, we found that the timing of reoperation was not associated with time-to-death following second surgery. A longer time to second surgery does not necessarily predict a commensurate longer time to death following second surgery, compared to a patient with a shorter time to their second surgery. The interpretation of this is that patients whose second surgery occurs later in the course of the disease are likely to survive longer overall, but not because of an additional survival benefit from the second surgery. This is consistent with the interpretation of Zhao et al. meta-analysis that demonstrated no survival benefit following second surgery once time to second surgery was controlled for [12]. Surprisingly, perhaps, almost two thirds of questionnaire respondents did not think that timing from first surgery mattered.

There may be merit in a minimum recommended time window from first surgery before consideration of further surgery, in the context of disease progression. Should this be time elapsed from the actual surgery, or from completion of chemotherapy? If the latter, would it be different for patients whose chemotherapy ended because of toxicity rather than disease progression? In the ongoing RESURGE study of surgery for progressive GBM, (https://www.resurge-trial.ch) eligibility includes first progression within 3 months of completion of radiotherapy, which broadly equates to only 4-5 months post-surgery. That study has recruited fewer than 40 patients in 5 years, underlining the challenges of recruiting to a randomised trial. The UK cohort data, with median time to second surgery of 13 months, suggests a preference amongst surgeons for a longer interval. Further, the questionnaire responses indicated that in some cases MDT referral was only made if survival since diagnosis was more than 9 months. Nine months post-surgery may be a useful cut-off for considering whether second surgery is likely to be beneficial. More evidence is needed thought to assess whether this is best for patients and since median progression free survival following standard care Stupp therapy is approximately 7 months [16], a shorter time interval may be appropriate to consider.

We cannot yet reliably predict who is most likely to benefit from second surgery. Development of probabilistic models may support treatment decision making. Scoring systems proposed to date have been developed typically from small patient cohorts (approx. 100), from analysis of retrospective data sets of patients who have had surgery, without accounting for the outcomes of people who don’t have second surgery. Factors in those models have included age, KPS, and tumour volume. More than 50% of questionnaire respondents thought surgery was only appropriate if the expected extent of resection was greater than 90%. The RESURGE study specifies inclusion criteria to target patients eligible for maximum resection, with complete resection of the contrast enhancing tumour considered feasible and no involvement of eloquent areas. Gross total resection (GTR) at first surgery has been consistently associated with improved patient outcomes, although the quality of the supporting evidence has been judged as moderate to low [17]. Support for the benefit of GTR second surgery has been reported [18, 19]. The DIRECTOR study compared TMZ regimens in GBM progression and a subgroup analysis of that study reported a significant increase in post-progression survival in patients having GTR [20], although those who had only partial resection tended to do worse than those without surgery (p = 0.52). This was a retrospective analysis of a selected study cohort, so the findings may not be generalisable. Moreover, predicted extent of tumour resection based on an individual patient’s imaging is contentious and can vary between surgeons, although scoring systems, artificial intelligence analysis and cloud-based systems for consensus gathering may help [21, 22]. Importantly, subtotal resection may still improve patient symptoms, and patients could potentially benefit from relatively modest debulking where recent tumour tissue for molecular analysis is required for entry into a clinical trial.

Whatever the surgical goals, maintenance or improving of quality of life are paramount; 90% of our questionnaire respondents agreed. This needs to be discussed with patients and to inform the decision whether to proceed with surgery. Success at maintaining and maximising quality of life depends in some part on the performance status of patients. 40% of questionnaire respondents suggested patients should have a KPS of at least 70 (caring for self but not capable of normal activity or work) for consideration of further therapy on progression, whilst 30% thought KPS should be higher. Surgery itself must not harm patients, but it is possible that surgery improves performance status through relief of mass effect, so KPS of less than 70 will not necessarily be a barrier to surgery. The incorporation of KPS score into a treatment decision making tool must also acknowledge inter-rater variation in assessment of an individual patient.

When tumour progression occurs, assessment of a patient’s function may be influenced by whether they are taking steroid medication. Our questionnaire and cohort study did not investigate the role of steroids specifically. Steroid use at progression has been suggestive as predictive of poor outcome and in an analysis of data from the BRAIN study of Bevacizumab and irinotecan in GBM progression, patients who were able to reduce their steroid therapy whilst on treatment had a better outcome [23]. However, surgery and tumour debulking may reduce a patient’s need for steroids, so functional dependence on dexamethasone may in fact be an indication for second surgery and predict good surgical outcome.

Alongside pre-operative performance status, age has also been reported as an important predictor of outcome at GBM progression [24, 25]. In that analysis of 333 phase I-II trial patients with GBM or malignant glioma, age less than 50 was associated with better outcomes, although those studies were performed before the advent of current standard care therapies. Median age of patients undergoing surgery in our UK cohort was 51, consistent with other published cohorts of patients at time of second surgery [26]. This is much younger than the average age of GBM diagnosis generally, and discordant with questionnaire responders, 45% of whom thought age was not important, and 24% of whom thought age over 70 should be the cut off.

Molecular tumour characteristics are playing an increasing role in the classification of gliomas, and treatment selection (ref; WHO, methylome). Brandes and colleagues reported a cohort of 270 patients who had second surgery for GBM progression, 44% of whom had methylation data [26]. Time from second surgery to death was significantly longer in people with methylated tumours; 13.8 v 10 months. Few of our questionnaire responders identified a role for MGMT methylation status in decision making, but this may change with increasing availability of the test. In our retrospective cohort study, 37.5% of patients who had repeat surgery had MGMT data available. Only 55% of these patients had promoter methylation, suggesting that MGMT methylation was not an important determinant of the decision to proceed to surgery. The lack of MGMT data likely reflects that it was not routinely available in these centres during the period of study, rather than that there was a bias for it to be tested in certain patients. In future analyses molecular correlates of outcome require more interrogation, and we may need to consider reports that molecular status can change at progression.

Some commentators question whether second surgery for GBM progression has a role at all, citing a lack of additional benefit beyond re-irradiation and chemotherapy [27, 28]. That does not though preclude that some patients can benefit from surgery. There may also be additional benefits when surgical adjuncts (e.g. 5-Aminolaevulinic acid, intra-operative MRI) are used, which were not systematically employed in our cohort, and require further assessment. The potential benefit of surgery in disease progression may also be linked to the opportunity for a patient to access other therapies, whether standard care or as part of a clinical trial. Repeat tissue sampling could be used to confirm marker expression for selection of targeted therapy, or development of the therapy itself, for example in vaccine trials [29]. Surgical debulking may support drug delivery or reduction of steroid dose, in turn improving efficacy of therapies such as immunotherapy [30]. 40% of responders to our questionnaire would consider repeat surgery even if no other treatments were available. Most commonly, however, PCV (49%) or re-challenge with TMZ if the tumour is methylated (42%) were preferred. This reflects the predominance of UK and European responders to our questionnaire. Single agent Lomustine at progression is most commonly used in North America and is the most common comparator for novel agents in randomised controlled trials of progression [31]. Any surgical trial will need to account for or control these variations in practice. In our surgical cohort only 22% of patients for whom there was data received TMZ after surgery and 11% PCV. In Brandes’ analysis, 34% received Temozolomide, 27% nitrosurea based therapy and 20% other therapies, but there was no difference in survival according to whether chemotherapy was delivered after second surgery, or what type of therapy was given [26].

## Conclusions

Our analysis is the most wide-ranging examination of contemporaneous practice in management of people with GBM progression. Clinician decision making is heterogeneous, yet there are trends from our analysis that could inform consensus guidelines (Table 3) This analysis provides a framework for further discussion amongst the neuro-oncology community, and to identify the priority areas for further research. Guidelines will not mandate selection for or against surgery. Rather, by identifying these patient factors as important in predicting benefit from second surgery, we hope to reduce heterogeneity in decision making, contribute to development of a data set in which these associations can be more intimately probed, and inform design of clinical trials. The role of surgery, surgical adjuncts, and decision-making tools are best addressed in prospective clinical studies, because heterogeneity in routine care, and the relative infrequency of second surgery, contribute to difficulties in curating adequately detailed and powered retrospective data sets, with appropriate comparators.

**Table 3:** Suggested criteria for consideration of second surgery

Criteria	Value
Recommend	
MRI T1 post-contrast, Perfusion or PET scanning may be required in some cases	Incontrovertible evidence of disease progression
Discussed at MDT	100% of patients
Clinical Objective	Improve Quality of Life
KPS > 70	≥ 70
Time from first surgery	≥ 9 months
Target extent of resection	≥ 90% contrast enhancing tumour
Desirable	
Availability of other therapies	Other therapies available
Comorbidities	Few

## Supplementary Information

Below is the link to the electronic supplementary material.

Supplementary material 1 (DOCX 325.3 kb)

## Data Availability

Requests for access to anonymised data should be made to the corresponding author.
