# Supplementary material for: Second surgery for progressive glioblastoma: a multi‐centre questionnaire and cohort‐based review of clinical decision‐making and patient outcomes in current practice
Source: J Neurooncol. 2021 Mar 31;153(1):99–107. doi: 10.1007/s11060-021-03748-0 (PMC8131335; doi:10.1007/s11060-021-03748-0)
Supplement: Supplementary file 1 — Supplementary material 1 (DOCX 325.3 kb) [file 11060_2021_3748_MOESM1_ESM.docx]

Neurosurgical Resection of First Recurrence of Glioblastoma

Questionnaire regarding your current practice for patients with recurrent glioblastoma and the ideal candidate for re-operation.

- 1. **Email address ***
  2. **Where are you based (city, country)? ***
  3. **What is your specialty? ***

*Mark only one oval.*

Neurosurgery - neuro-oncology sub-specialist Neurosurgery - not neuro-oncology sub-specialist Clinical Oncology

Medical Oncology Palliative Care Neurology Radiology

Other:

- 1. **Approximately how many craniotomies for glioblastoma are performed in your Hospital, annually? ***

*Mark only one oval.*

0

1-20

21-50

51-100

>100

- 1. **How many resections for first recurrence of glioblastoma are performed each year in your hospital? ***

*Mark only one oval.*

0

1-3

3-5

5-10

>10

- 1. **Following completion of Stupp protocol treatment, what is the approximate frequency of MRI surveillance in the FIRST year?**

*Mark only one oval.*

3-4 monthly.

6 monthly Anually

Upon symptomatic recurrence

Other:

- 1. **Following completion of Stupp protocol treatment, what is the approximate frequency of MRI surveillance in the SECOND year?**

*Mark only one oval.*

3-4 monthly.

6 monthly Anually

Upon symptomatic recurrence

Other:

- 1. **Are all glioblastoma recurrences reviewed at your MDT? ***

*Check all that apply.*

Yes

Local recurrences only

Good performance status only

Patients who have survived more than 9 months since surgery Please add any other discriminators that you use under 'other' Other:

- 1. **What radiological criteria would your MDT use to diagnose a recurrent glioblastoma and determine a plan to proceed to re-do surgery? (please tick all that apply) *** *Check all that apply.*

MRI - Local recurrence of contrast enhancing disease. Magnetic resonance Spectroscopy

Magnetic resonance Perfusion

PET (please state isotope under 'other' as free text) Other:

- 1. **Is there an approximate maximum age at the time of recurrence that makes a patient UNsuitable for re-do surgery (not withstanding other molecular or tumour features)? ***

*Mark only one oval.*

Age does not influence decision making

>40 years

>50 years

>60 years

>65 years

>70 years

>80 years

- 1. **Does the methylation status of the MGMT promoter matter in tumour from the first surgery? ***

*Check all that apply.*

We don't routinely test MGMT methylation status MGMT methylation status does not matter

MGMT methylation is an indicator to proceed to recurrent surgery Absence of MGMT methylation is a marker AGAINST recurrent surgery

- 1. **Is there a minimum KPS score for a patient at the time of recurrence to be considered for resection? ***

*Mark only one oval.*

No KPS preference 90-100

80

70

Other:

- 1. **Would you consider recurrent surgery in a patient who is progressing while still on treatment? ***

*Mark only one oval.*

Yes, tumour progression BEFORE completion of ADJUVANT chemotherapy precludes re-do surgery

No preference

- 1. **Does the anticipated extent of resection (in % of contrast enhancing tumour) influence your decision to recommend resection for recurrent glioblastoma? ***

*Mark only one oval.*

No, the anticipated extent of resection does not influence my decision. I select patients expected to have a 'total' resection (100%)

I select patients expected to have at least 90% resection

I select patients expected to have at least 80% resection I select patients expected to have at least 50% resection

- 1. **Which of the following is most important in considering suitability for resection of recurrent glioblastoma?**

*Mark only one oval.*

Total time after first surgery?

Total time after completion of chemotherapy ? (which will vary according to number of cycles of chemotherapy completed).

- 1. **Is there a minimum duration of time after the primary resection that must pass before you consider resection of recurrent tumour? ***

*Mark only one oval.*

No

Yes, 2 Months

Yes, 4 Months

Yes, 6 Months

Yes, 9 Months

Yes, 12 months. Other:

- 1. **Should a decision for recurrent surgery only be made if the oncologists are prepared to consider further treatment (re-irradiation or chemo) post-operatively? ***

*Mark only one oval.*

Yes No

- 1. **On identification of recurrent tumour suitable for surgery:**

*Mark only one oval.*

The patient would be recommended to proceed to surgery on the next available list (within 7-14 days)

We would repeat imaging in 4-6 weeks to assess speed of change Other:

- 1. **Which of the following chemotherapy options are used after recurrent surgery in your unit if already post radiotherapy and temezolomide?**

*Check all that apply.*

PCV

Temozolomide if methylated at first surgery Temozolomide regardless of methylation status Single agent lomustine

Other:

- 1. **Is there a role for re-irradiation of patients who have previously completed concomitant chemo-radiotherapy followed by adjuvant chemotherapy (Stupp protocol)?**

*Mark only one oval.*

Yes No

Yes, but only in a clinical trial Other:

- 1. **Are there any other considerations you have prior to considering a patient for resection of recurrent glioblastoma?**

*Mark only one oval.*

Quality of life Other:

- 1. **Would you be interested in being involved in a multi-centre randomized controlled trial to ascertain the efficacy of resection of recurrent glioblastoma? ***

*Mark only one oval.*

Yes No

- 1. **If you answered yes, please leave your email address so that we can contact you**
